# Weighted Kernel Entropy Component Analysis for Fault Diagnosis of Rolling Bearings

**DOI:** 10.3390/s17030625

**Published:** 2017-03-18

**Authors:** Hongdi Zhou, Tielin Shi, Guanglan Liao, Jianping Xuan, Jie Duan, Lei Su, Zhenzhi He, Wuxing Lai

**Affiliations:** 1 State Key Laboratory of Digital Manufacturing Equipment and Technology, Huazhong University of Science and Technology, Wuhan 430074, China; zh_hongdi@hust.edu.cn (H.Z.); tlshi@hust.edu.cn (T.S.); jpxuan@hust.edu.cn (J.X.); jduan@hust.edu.cn (J.D.); 2 School of Mechanical Engineering, Jiangnan University, Wuxi 214122, China; lei_su2015@jiangnan.edu.cn; 3 School of Mechanical & Electrical Engineering, Jiangsu Normal University, Xuzhou 221116, China; hezz82@163.com

**Keywords:** fault diagnosis, weighted kernel entropy component analysis, dimensional reduction, Renyi entropy, feature extraction

## Abstract

This paper presents a supervised feature extraction method called weighted kernel entropy component analysis (WKECA) for fault diagnosis of rolling bearings. The method is developed based on kernel entropy component analysis (KECA) which attempts to preserve the Renyi entropy of the data set after dimension reduction. It makes full use of the labeled information and introduces a weight strategy in the feature extraction. The class-related weights are introduced to denote differences among the samples from different patterns, and genetic algorithm (GA) is implemented to seek out appropriate weights for optimizing the classification results. The features based on wavelet packet decomposition are derived from the original signals. Then the intrinsic geometric features extracted by WKECA are fed into the support vector machine (SVM) classifier to recognize different operating conditions of bearings, and we obtain the overall accuracy (97%) for the experimental samples. The experimental results demonstrated the feasibility and effectiveness of the proposed method.

## 1. Introduction

Rolling element bearings are widely used in rotating machines in modern industry, and bearing failure is one of the most common reasons for machine breakdown. Unexpected failures may cause huge economic losses and even lead to casualties [1,2,3]. Therefore, it is important to accurately diagnose bearing faults at the early stage [4,5]. Vibration-based fault diagnosis has been extensively studied to improve existing techniques toward the goal of more accurately dealing with various problems, such as varying load effect and noise contamination [3,4,5,6,7,8]. Especially, the sensitivity of diagnostic features from the vibration signals may vary with different load conditions due to nonlinear effect and non-stationary noise, of which no single-domain processing methods can comprehensively extract the fault features to reflect the condition [9]. High-dimensional feature sets constructed with mix-domain features are often used for diagnosis [10,11]. Although more features can obviously provide more information, they contain a lot of redundant and disturbed information which will increase computation time and reduce recognition accuracy. More effective feature extraction and dimensionality reduction methods are needed to obtain higher diagnostic accuracy [12,13].

Principal component analysis (PCA) is one typical method for dimensionality reduction and has been widely used for fault diagnosis [14,15,16,17], since it can extract representative features from high-dimensionality, noisy and linear correlated data. PCA is an unsupervised method that projects the original dataset onto a lower-dimensional space meanwhile minimizes the mean square error [15]. It can guarantee that the linear features can be extracted while some useful nonlinear features may be lost, as the most of industrial systems are non-linearity and non-stationary. Therefore, nonlinear methods are required to handle the nonlinear data, among which kernel principal component analysis (KPCA) [18] is the most prominent one. KPCA is an extension of traditional linear PCA by using kernel trick, implicitly mapping the original features into a high-dimensional feature space in which the mapped data are linearly separable and then the linear PCA can be conducted [15]. Both PCA and KPCA are typical spectral dimensionality reduction methods which extract features by selecting the top eigenvalues and corresponding eigenvectors of the specially constructed feature matrixes [19]. Hence, the extraction may select uninformative eigenvectors from the information theory standpoint [20].

Kernel entropy component analysis (KECA) is a newly developed information-theory-based dimensionality reduction method, first proposed and employed in pattern recognition by Robert Jenssen [21]. This method attempts to maintain the maximum estimated Renyi quadratic entropy of the input data set via a kernel-based estimator. It is fundamentally different from other methods in two ways: on the one hand, the selection of top eigenvalues and corresponding eigenvectors is not necessary; on the other hand, the dimension reduction reveals the intrinsic structure related to the Renyi entropy of the input data [21,22,23,24,25]. Moreover, KECA typically generates a transformed dataset with a distinct angular structure, implying that even nonlinearly related input data sets are distributed in different angular directions with respect to the high-dimensional kernel feature space [21,22,23,24,25]. KECA has been applied to feature extraction and pattern recognition successfully, showing superior performance over PCA and KPCA [21,22,23,24]. However, KECA is unsupervised, ignoring the label information of the input data, which may discard discriminant classification information and weaken recognition accuracy [25]. And the projections in PCA, KPCA and KECA are theoretically optimal for reconstruction from a low-dimensional basis, while they may not be optimal from the viewpoint of discrimination. Many previous studies attempt to extract discriminative features to express the original clusters [25,26], and meanwhile to find a trade-off between maximizing the testing accuracy and minimizing the training error [20,26,27].

In this study, we propose a supervised feature extraction method called weight kernel entropy component analysis (WKECA) based on KECA, in which a modified Fisher criterion is applied to represent class separability. The class-related weights are introduced to denote differences among the samples from different patterns, and genetic algorithm (GA) is applied to seek out appropriate weights for optimizing the classification results. Experimental investigation is conducted to demonstrate the feasibility and effectiveness of the proposed method for fault diagnosis.

## 2. The Theoretical Background of WKECA for Fault Diagnosis

### 2.1. Brief Review of KECA

Assuming that *p*(**x**) is the probability density function of a given sample **X** = **x_1_**, …, **x_N_**, its Renyi entropy of the order *α* is expressed as $H_{\alpha }\left(X\right)=\frac{1}{1-\alpha }\mathrm{lg}\left(\int p^{\alpha }\left(x\right)dx\right)$ [28], where *α* ≥ 1. In KECA, Renyi quadratic entropy (*α* = 2) is employed, because the entropy value can be elegantly estimated by Parzen window density estimator [29]. Renyi quadratic entropy can be expressed by $H\left(X\right)=-\mathrm{lg}\left(\int p^{2}\left(x\right)dx\right)$. Since the monotonicity property of logarithmic function, only the integral function $V\left(p\right)=\int p^{2}\left(x\right)dx=E\left\{p\left(x\right)\right\}$ needs to be considered [21,22,30]. To estimate *V*(*p*), a Parzen window density estimator $\bar{p}\left(x\right)=\frac{1}{N}\sum_{x_{i}\subset D}K_{\sigma }\left(x,x_{i}\right)$ is applied [21,29], where ***K_σ_***(**x**, **x_i_**) is the estimator or kernel function centered at **x_i_** and *σ* is the smoothing width or the kernel size. According to the convolution theorem, the convolution of two Gaussian functions is another Gaussian function with $\sigma =\sqrt{{\sigma_{1}}^{2}+{\sigma_{2}}^{2}}$. Substituted *K**_σ_***(**x**, **x_i_**) and $\bar{p}$(**x**) into *V*(*p*), the following estimation can be obtained:

(1) $$\begin{array}{ll} \bar{V}\left(p\right) & =\int p^{2}\left(x\right)dx=\frac{1}{N^{2}}\sum_{i=1}^{N}\sum_{j=1}^{N}\int K_{\sigma }\left(x,x_{i}\right)K_{\sigma }\left(x,x_{j}\right)dx\\ & =\frac{1}{N^{2}}\sum_{i=1}^{N}\sum_{j=1}^{N}K_{\sqrt{2}\sigma }\left(x_{i},x_{j}\right)=\frac{1}{N^{2}}1K1^{T} \end{array}$$

where **K** is a N × N kernel matrix, the element (i, j) of **K** is *K_σ_*(**x_i_, x_j_**), and **1** is a N × 1 vector (all elements are one). Therefore, the Renyi entropy can be estimated by the corresponding kernel matrix that can be decomposed as **K** = **EDE^T^**, where **D** = *diag*(*λ*_1_, *λ*_2_,..., *λ*_N_) and **E** = [**α_1_**, **α_2_***,...,***α_N_**]. Here *λ*_i_ and **α_i_** are the eigenvalues and corresponding eigenvectors, respectively. Then:

(2) $$\bar{V}\left(p\right)=\frac{1}{N^{2}}1K1^{T}=\frac{1}{N^{2}}1EDE^{T}1^{T}=\frac{1}{N^{2}}\sum_{i=1}^{N}{\left(\sqrt{\lambda_{i}}{\alpha_{i}}^{T}1\right)}^{2}$$

This expression is the so-called entropy values, and each term $\sqrt{\lambda_{i}}{\alpha_{i}}^{T}$ contributes to the entropy estimation. The eigenvectors and corresponding eigenvalues are ranked in decreasing order of the entropies. KECA selects certain eigenvalues and corresponding eigenvectors according to the *d* largest entropies [21], different from PCA and KPCA that select largest eigenvalues. Therefore, the resulting KECA expression is $\Phi_{keca}={D_{d}}^{\frac{1}{2}}{E_{d}}^{T}$, where **D_d_** and **E_d_** store the top d eigenvalues and corresponding eigenvectors.

### 2.2. Introduction of WKECA

Given a set of *c*-class training sample patterns $x_{i}\in R^{N}$ (i = 1, 2, ..., N), and each sample **x_i_** belongs to one of *c*-class. Defined that the weight vector is [*u*_1_, *u*_2_, ..., *u*_N_] and the label values are {*l*_1_, *l*_2_, ..., *l_c_*}. Each sample has the corresponding label value based on its own class properties. Thus, *u*_i_ *= l*_j_ if $x_{i}\in$ j-th class, where i = 1, 2, ..., N and j = 1,2, ..., *c*. Here the weights are depended on the class so that they can represent the class information. The weighted matrix that has the same dimension as the original kernel matrix *K*(**x_i_**, **x_j_**) is defined as:

(3) $$W\left(i,j\right)=\langle \Phi \left(u_{i}\right),\;\Phi \left(u_{j}\right)\rangle =\exp\left(-\frac{{\left\|u_{i}-u_{j}\right\|}^{2}}{2\sigma^{2}}\right)$$

We constructed the new weighted kernel matrix **K_w_** with $K_{W}\left(i,j\right)=K{\left(i,j\right)}^{W\left(i,\;j\right)}$ as:

(4) $$K_{W}\left(i,\;j\right)=K{\left(i,\;j\right)}^{W\left(i,\;j\right)}=\exp\left(-\frac{{\left\|x_{i}-x_{j}\right\|}^{2}\exp\left(-\left({\left\|u_{i}-u_{j}\right\|}^{2}\right)/2\sigma^{2}\right)}{2\sigma^{2}}\right)$$

The effects of the weights under two conditions can be analyzed: (1) If *u*_i_ = *u*_j_, the samples **x_i_** and **x_j_** belong to the same class and *W*(i, j) = 1. As observed, the weighted kernel matrix **K_W_** will be equal to the original kernel matrix **K**. (2) If *u*_i_ ≠ *u*_j_, the *W*(i, j) will be a positive value, in which the label information can be embedded in the weighted kernel matrix.

Eigen-decomposed **K_W_**: **K_W_** = **E_W_D_W_E_W_^T^**, the eigenvalues *λ*_w1_, *λ*_w2_, ..., *λ*_wN_ of the weighted kernel matrix are ranked in decreasing order of the entropies, and **α_w1_**, **α_w2_**, ..., **α_wN_** are the corresponding eigenvectors. The subspace is defined as **U_W_** spanned by the principal axes that contribute most to the Renyi entropy estimation. Requiring ||**u_wi_** ||^2^ = 1, thus **u_wi_** = *λ*_wi_**Φα_wi_** can be obtained. We can project both training and testing samples onto **U_W_** to extract the intrinsic features. For the out-of-sample data set **x_t_**, the extracted features can be calculated:

(5) $$\begin{array}{cc} y(x_{t} & )={u_{wi}}^{T}\Phi \left(x_{t}\right)=\langle U_{W},\Phi \left(x_{t}\right)\rangle =\langle {\lambda_{\mathrm{wi}}}^{-\frac{1}{2}}\sum_{i=1}^{N}\alpha_{wi}\Phi \left(x_{i}\right),\Phi \left(x_{t}\right)\rangle \\ & ={\lambda_{\mathrm{wi}}}^{-\frac{1}{2}}\sum_{i=1}^{N}\alpha_{wi}k_{\sigma }\left(x_{i},x_{t}\right)={D_{W}}^{-\frac{1}{2}}E_{W}{}^{T}K^{\prime^{T}} \end{array}$$

Let $\Phi^{\prime }$ refer to a collection of the out-of-sample data sets, $K^{\prime }=\Phi^{\prime^{T}}\Phi$ is the inner product matrix. Then we can extract the first d nonlinear principal components which contribute most to Renyi entropies of the input data by using the weighted kernel matrix. The number, d, of the projection vectors is determined in terms of $\sum_{i=1}^{d}{\left(\sqrt{\lambda_{i}}\alpha_{i}{}^{T}1\right)}^{2}/\sum_{j=1}^{N}{\left(\sqrt{\lambda_{j}}\alpha_{j}{}^{T}1\right)}^{2}\geq \alpha$ (set to 0.95 here for both KECA and WKECA).

### 2.3. Selecting Optimal Weights for Weighted Kernel Entropy Component Analysis by Genetic Algorithm

The relevance of different classes leads to diversified generalization performances. Therefore, weights are important to the recognition system, and determination of weights can be considered as an optimization problem. GA is a search and optimization process inspired by the laws of nature evolution and selection [31], which is a powerful intelligent optimization tool based on a group of independent computations controlled by the probabilistic strategy. GA has been widely used in various applications because of its excellent global search ability [31,32]. In this study, we use GA to find the most suitable weights for WKECA where the optimality is defined regarding the recognition accuracy and class separability. The main optimization process can be described as follows:

(1) Individual encoding: defined the individual is a set of weights *l*_1_, *l*_2_, ..., *l_c_*, the encoding method based on binary for each weight is used.
(2) Population initialization: an initial population with *n_r_* individuals (set to 20) is randomly created.
(3) Fitness calculation: the individual selection for the next generation is done based on the fitness. Taking advantage of Liu and Wang’s work [19], the fitness function is defined as *f*(*X*) = CA + *kR_BW_*, where CA is the training accuracy which can represent the performance of extracted features, *k* is a positive constant, and *R_BW_* is the Fisher criterion which can indicate the class separability. *R_BW_* is the ratio of between-class distance *S_b_* and within-class distance *S_w_* [33]. High classification accuracy and large class separability can be obtained by maximizing the fitness function, which results in evolving more discriminate information than KECA with a proper *k*. Therefore, good generalization performance for WKECA is possible to be acquired on both training and testing samples.
(4) Genetic operators: new chromosomes are generated to update and optimize population continuously by genetic operators including selection, cross-over and mutation. The crossover probability and mutation probability are set to 0.7 and 0.01, respectively. The selected probability of every individual is $p_{m}=\frac{f\left(w_{m}\right)}{\sum_{m}^{n_{r}}f\left(w_{m}\right)}$, m = 1,... , *n_r_*, where *f*(*w_m_*) is the individual’ fitness value.
(5) Terminating conditions: when the value of fitness does not change again during the iteration procedure or the number of iterations has reached the maximum value (50 in this study) the program will terminate.

### 2.4. Fault Diagnosis Based on WKECA

The high-dimensional feature set, which can represent well the operating condition of machines, should be first extracted from the raw vibration signals. Generally, the vibration signals of fault bearings are non-stationary, and wavelet packet decomposition (WPD) that can provide a more meticulous analysis is a powerful tool in dealing with non-stationary signals [34]. WPD is effective for decomposing both high- and mid-frequency information from a signal into the corresponding frequency regions, widely used for fault diagnosis of bearings now [34,35,36,37,38]. In this study, WPD is performed to extract the fault features including the relative energy in a wavelet packet node (REWPN) and the entropy in a wavelet packet node (EWPN). The REWPN indicates the normalized energy of the wavelet packets node, and the EWPN represents the uncertainty of the normalized coefficients of the wavelet packets node [39]. For a given sample *x*(*n*), the *j*th wavelet packet coefficients of the *i*-th wavelet packet node is defined as $C_{i}{}^{j}$, and then REWPN and EWPN can be expressed as follows:

(6) $$\mathrm{REWPN}\left(i\right)=\frac{\sum_{j=1}^{K}{\left(C_{i}{}^{j}\right)}^{2}}{\sum_{m=1}^{N}\sum_{j=1}^{K}{\left(C_{m}{}^{j}\right)}^{2}}$$

(7) $$\mathrm{EWPN}\left(i\right)=-\sum_{j=1}^{K}p_{i}{}^{j}\log_{2}\left(p_{i}{}^{j}\right)$$

where $p_{i}{}^{j}={\left(C_{i}{}^{j}\right)}^{2}/\sum_{j=1}^{K}{\left(C_{i}{}^{j}\right)}^{2}$, *N* is the total number of wavelet packet nodes, and *K* is the total number of wavelet packet coefficients in each wavelet packet node.

**Figure 1:**
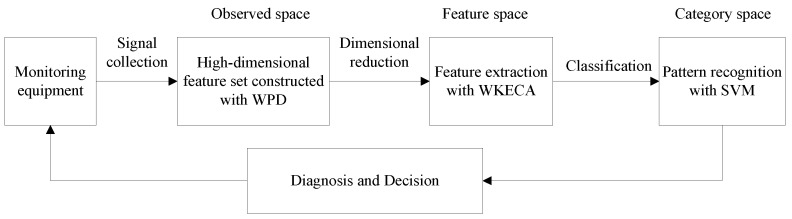
Implementation process of the proposed fault diagnosis method.

The REWPNs and EWPNs can truly reflect the diversity among different fault patterns of bearings. They are used as the high-dimensional input vector to WKECA for dimensionality reduction, which can be written as **x_i_** = [REWPN (1), ..., REWPN (*p*), EWPN (1), ..., EWPN (*p*)]^T^. Here, *p* is the number of wavelet packet node. The implementation process of the proposed fault diagnosis method using WKECA for bearings is detailed as shown in Figure 1:

(1) Decomposing the vibration signals into different frequency bands by using WPD, and then we can acquire the high dimensional feature set **X** = [**x**_1_, ..., **x**_N_]^T^ including REWPNs and EWPNs, where *N* is the number of the signal samples.
(2) Carrying out feature extraction to the high-dimensional dataset obtained from vibration signals with WKECA algorithm, capturing their intrinsic manifold structure, and then we can obtain the low-dimensional features by projecting the original high-dimensional observed space into low-dimensional feature space. Meanwhile, the optimal mapping direction can be acquired so that new testing samples can be mapped into the low-dimensional feature space.
(3) Implementing pattern classification of the datasets in the low-dimensional feature space with support vector machine (SVM) classifier.
(4) Determining the type of failures by the classification results, and we can put forward the corresponding decisions or control measures.

## 3. Experimental Results and Analysis

### 3.1. Experimental Description

**Figure 2:**
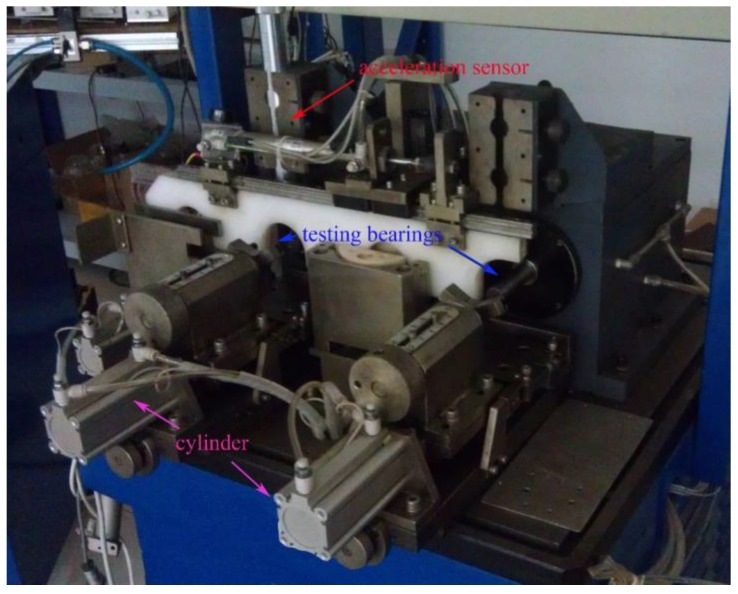
The test rig.

To evaluate the effectiveness of the WKECA, an experimental study on fault diagnosis of rolling bearings was performed. As shown in Figure 2, the tested bearings were delivered through the automatic machinery system which contained the preset mechanism, the measuring mechanism, the sorting mechanism, and the feeding mechanism [40,41]. The radial vibration signals on one point of the tested bearings were detected by a piezoelectric acceleration sensor (YD-1, Far East Vibration (Beijing) System Engineering Technology Co., Ltd., Beijing, China) located on the top of the bearings, and amplified by a charge amplifier (DHF-2, same company as the sensor). The charge sensitivity and frequency response of the sensor are 6–10 pC/ms^−2^ and 1–10,000 Hz ± 1 dB, respectively, and the frequency range of the amplifier is 0.3 Hz–100 kHz. Then the signals were converted to voltage signals by an A/D converter (PCI-9114) (ADLINK Technology, Inc., Taiwan) and sent to a computer for further processing. The sampling frequency was 25 kHz, and the rotational speed of the driving motor was set to 1500 rpm.

**Figure 3:**
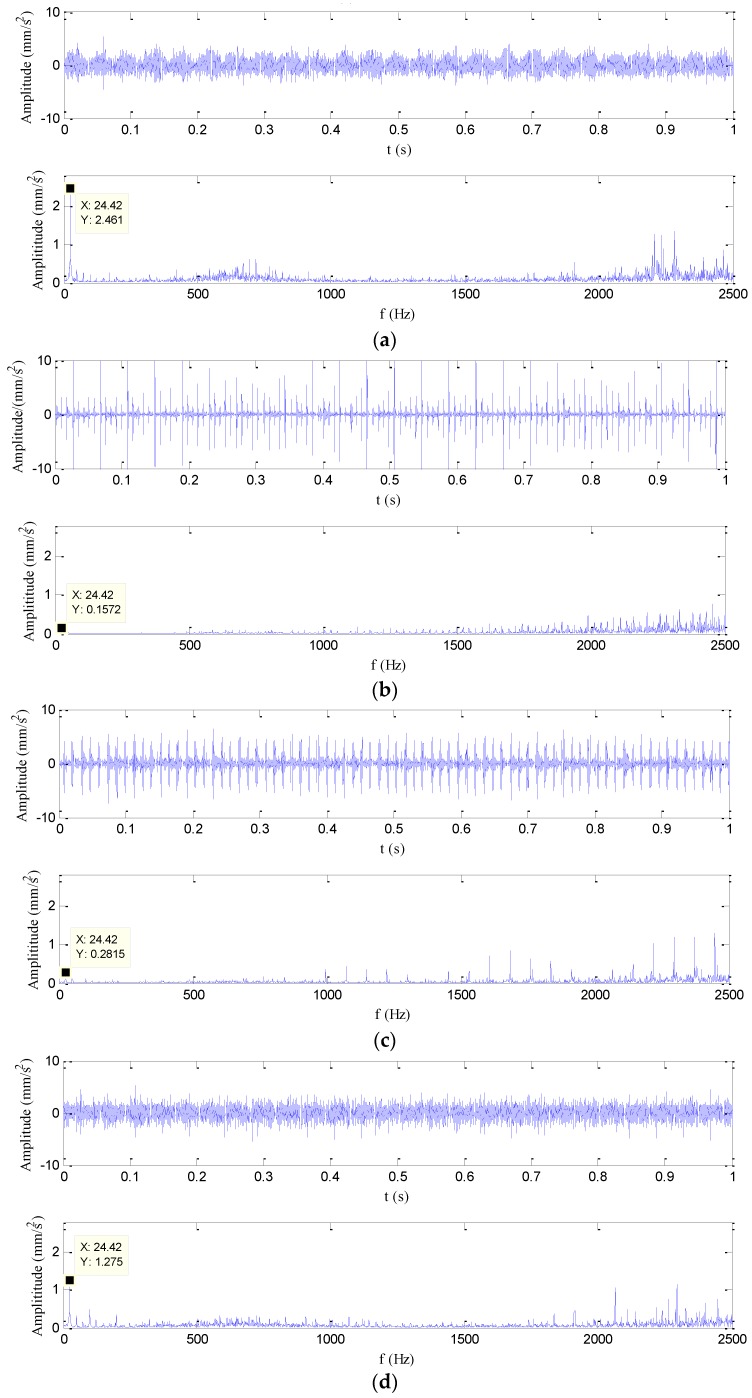
The time domain and frequency domain figures of vibration signals for the four bearing conditions: (**a**) normal condition, (**b**) inner race fault, (**c**) outer race fault, and (**d**) ball fault.

Deep groove ball bearings (6328-2RZ) (Changjiang bearing co., LTD, Chongqing, China) were used as the tested bearings, and four different operating conditions (i.e., inner race fault, outer race fault, ball fault, and normal condition) were simulated in this experiment. Single point defects were introduced to the tested bearings by electric engraving pen, where the widths of the scratch defects were 65 ± 22 μm, 70 ± 20 μm, and 70 ± 20 μm for the inner race, outer race and ball, respectively, and the depths of the scratch defects were 0.2 ± 0.05 mm. The characteristic bearing defect frequencies can be calculated by [42]:

(8) $$\mathrm{Defect}\;\mathrm{on}\;\mathrm{inner}\;\mathrm{race}\;(\mathrm{BPI})\;=\;\frac{Zf_{r}}{2}(1+\frac{d}{D}\cos\alpha )$$

(9) $$\mathrm{Defect}\;\mathrm{on}\;\mathrm{outer}\;\mathrm{race}\;(\mathrm{BPO})\;=\;\frac{Zf_{r}}{2}(1-\frac{d}{D}\cos\alpha )$$

(10) $$\mathrm{Defect}\;\mathrm{on}\;\mathrm{ball}\;(\mathrm{BS})\;=\;\frac{f_{r}D}{2d}(1-\frac{d^{2}}{D^{2}}\cos^{2}\alpha )$$

where *Z* is the number of rolling elements, *f_r_* is the rotational frequency, *d* is the diameter of the rolling element, *D* is the pitch diameter, and α is the contact angle. According to the kinematic parameters of the tested bearings and the rotational speed, the characteristic bearing defect frequencies of the inner race, outer race and ball are 121.75 Hz, 78.25 Hz and 55 Hz, respectively. Figure 3 indicates the four different vibration signal waveforms in the time-domain together with the amplitude spectrums. The peak values of the accelerations are obtained at 24.42 Hz which is closed to the rotational frequency 25 Hz. As observed, it is difficult to distinguish different faults only from Figure 3 due to the effects of the noise. The vibration signals under those four conditions are selected as samples, and 100 bearings for each state were tested. Thus, 400 data can be obtained, and the length of each data set is 25,000. The training data set is half samples of the original data set in the experiment.

### 3.2. Dimensionality Reduction and Pattern Classification

**Figure 4:**
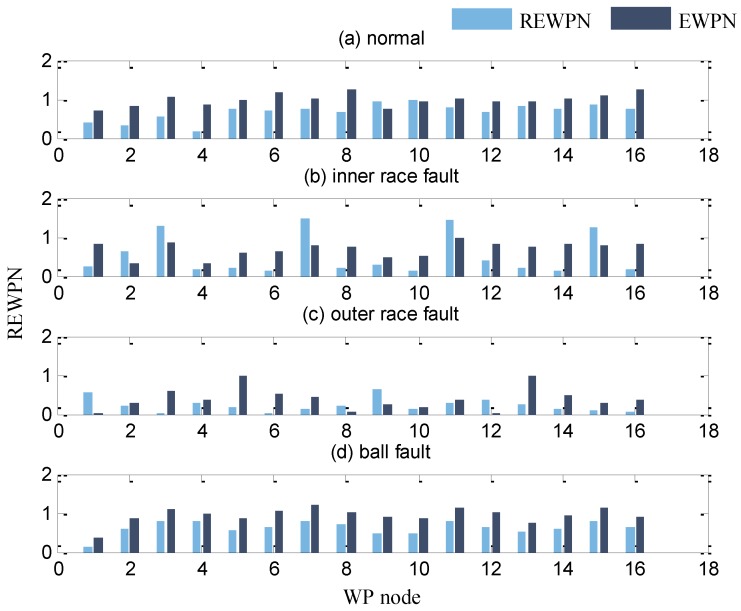
The normalized wavelet packet energy and entropy spectrums of the bearing vibration signals under four conditions: (**a**) normal condition, (**b**) inner race fault, (**c**) ball fault, (**d**) outer race fault.

The high dimensional feature set containing REWNs and EWPNs are first constructed. The wavelet packet node energy features obtained by Daubechies2 (db2) wavelet packet decomposition were found to achieve the best classification performance for bearing fault diagnosis after many experiments on a serials of Daubechies wavelets [43]. Here the Daubechies2 (db2) is selected as the mother wavelet function to implement binary WPD for vibration signals, where the maximum decomposition level is set to 4. The normalized wavelet packet energy and wavelet packets node entropy spectrums of the bearing vibration signals are shown in Figure 4. Obviously, different bearing faults have different amplitude in different frequency bands. 32 fault features in total including 16 REWPNs and 16 EWPNs are used for fault diagnosis of bearings.

**Figure 5:**
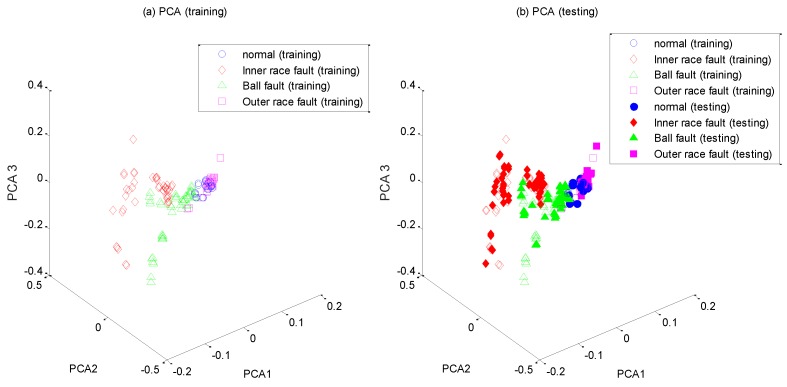
Feature extraction with PCA: (**a**) training samples, (**b**) testing samples.

**Figure 6:**
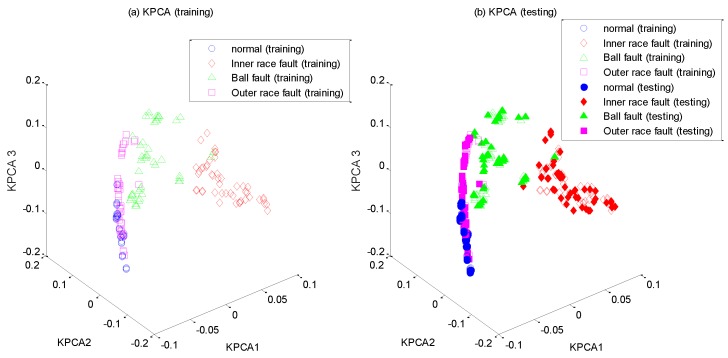
Feature extraction with KPCA: (**a**) training samples, (**b**) testing samples.

**Figure 7:**
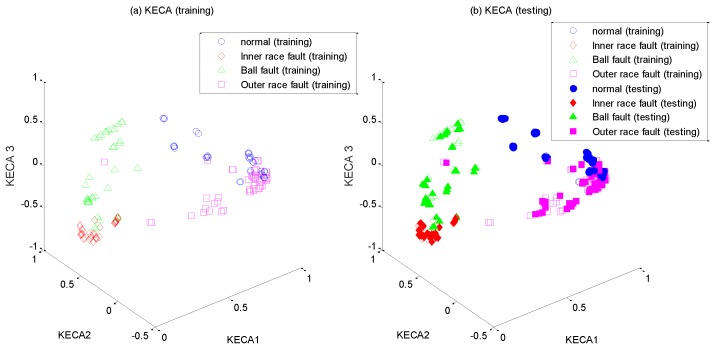
Feature extraction with KECA: (**a**) training samples, (**b**) testing samples.

**Figure 8:**
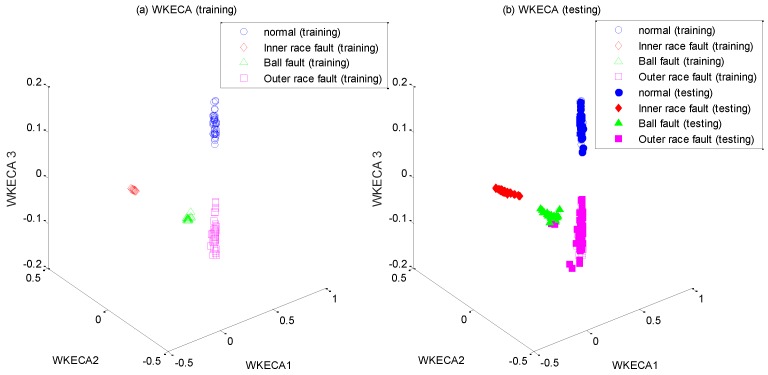
Feature extraction with WKECA: (**a**) training samples, (**b**) testing samples.

After the high-dimensional feature set is constructed, it is input into WKECA for non-linear dimension reduction, where the parameter *k* of the fitness function is set to 0.001. The first d most significant component vectors contributing most to the Renyi entropy are extracted by WKECA, and similar methods including PCA, KPCA and KECA are conducted for comparison. The target dimensionality for every method is set to a certain number so that the cumulative variance contribution rate is more than 95%. For visualization, the plots of the first three principal components of their projection results are shown in Figure 5, Figure 6, Figure 7 and Figure 8, where Figure 5a, Figure 6a, Figure 7a and Figure 8a represent the training results, and Figure 5b, Figure 6b, Figure 7b, and Figure 8b represent the testing results. It is evident that PCA, KPCA and KECA are not well separated those four classes because some samples are overlapped, which will lead to low recognition accuracy. By contrast, WKECA has little misjudgment samples: the testing points are consistent with the training points in WKECA, and the WKECA algorithm can obviously identify different classes both for the training samples and the testing samples. It proves that WKECA has better clustering performance than PCA, KPCA and KECA, because WKECA introduces the fault class label information and a weight strategy into feature extraction, which is conductive to pattern recognition.

### 3.3. Results and Discussion

Within the fault diagnosis related to pattern recognition in conjunction with feature extraction techniques that find low-dimensional representation for samples, classifiers are needed to identify those different bearing faults. Support vector machine (SVM) is adopted for its well-developed statistical learning theory. 50 data from inner race fault, outer race fault, ball fault, and normal condition were selected randomly for SVM training and the others were used for testing. The quantitative evaluation procedure for SVM, PCA-SVM, KPCA-SVM, KECA-SVM, and WKECA-SVM were repeated for 10 times. In order to highlight the effectiveness of the proposed WKECA-SVM method, the fault detection rate of the method was compared with the results of the other four methods. The testing average results are summarized in Table 1, and the classification accuracies are 77.5%, 83%, 89.5%, 93% and 97%. The results demonstrate that satisfactory overall classification results have been achieved by means of the dimension reduction, and the classification accuracy is significantly improved by introducing WKECA. WKECA performs better than the other methods in terms of extracting discriminative features which can lead to high classification rates. Therefore, WKECA is suitable as a feature extraction step prior to classification, and functions well for fault patterns recognition.

To obtain discriminative representations through GA, a suitable fitness function is important to the whole recognition procedure. Therefore, it is necessary to know the effects of the parameter *k* in fitness function. Table 2 presents the results of evolutionary process with different *k*, where CA_test_ is the testing accuracy. It is obvious that R_BW_ increases with the raising of *k* while CA_test_ decreases accordingly. This observation reflects that *k* can adjust the contribution of class separability to the fitness function, and a proper *k* can lead to larger R_BW_ as well as good classification performance.

**Figure 9:**
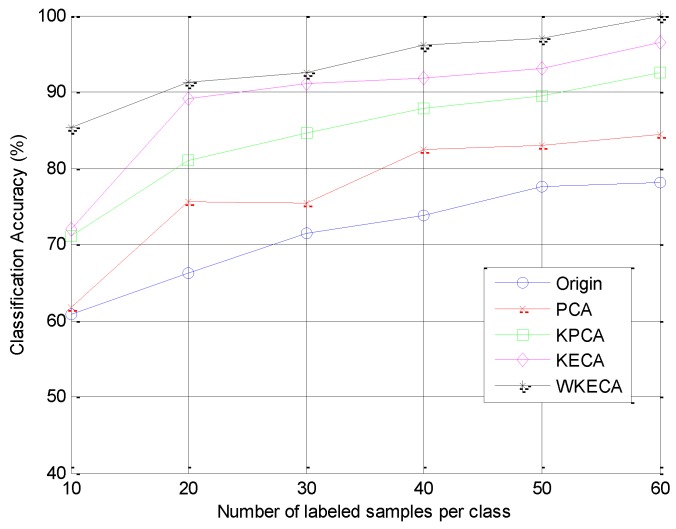
Classification accuracy of SVM based on different feature extraction methods for different labeled samples.

In order to investigate the performance of WKECA in handling the Small Sample Size (SSS) problem with different training sample sizes, PCA, KPCA and KECA were conducted for comparison. Figure 9 presents the recognition rates of the four feature extraction methods and the original features with different numbers of labeled samples. It is obvious that the classification accuracy increases with the raising of training sample sizes. This reveals that the feature extraction based on manifold learning can improve the recognition performance, and WKECA performs better than other methods in achieving high classification accuracy. The effects of SSS problem are obvious in other methods when only ten samples are used for training, while WKECA is less sensitive to the training sample size. This proves that WKECA can capture the intrinsic geometric structure embedded in the data and achieve efficient performance in feature extraction and classification.

## 4. Conclusions

In this study, a new feature extraction method called weighted entropy component analysis (WKECA) is proposed for fault diagnosis of rolling bearings. It makes the most of the labeled information and introduces a weight strategy in feature extraction, and GA is performed to find optimal weights for achieving high training classification results. The original high-dimensional feature sets are first constructed based on WPD which can provide a more meticulous analysis for signals. WKECA is then used to extract the intrinsic independent features among the multiple manifolds to reflect the states of the rolling bearings. Finally, the extracted intrinsic geometric features are fed into SVM to recognize different operating conditions of bearings. WKECA outperforms PCA, KPCA and KECA in terms of achieving higher testing accuracies. The results demonstrate the feasibility and effectiveness of the proposed method for fault diagnosis of rolling bearings. Next, we are trying to extend our approach to diagnose different faults magnitudes in different machines. The challenge is the great time consumption for training, which is inevitable confronted by almost all evolutionary processes for pattern recognition. Therefore, fast optimal strategies are deserved for further investigation.

## Figures and Tables

**Table 1:** The classification accuracies of different methods to the bearing sets with support vector machine (SVM) classifier.

Operating Condition	Normal (%)	Inner Race Fault (%)	Outer Race Fault (%)	Ball Fault (%)	Average Accuracy (%)
Original	68	86	76	80	77.5
PCA	72	90	88	82	83
KPCA	92	92	84	90	89.5
KECA	96	98	82	96	93
WKECA	100	100	92	96	97

**Table 2:** The results of evolutionary process with different values of parameter k.

Performance	*k* = 0.001	*k* = 0.01	*k* = 0.1	*k* = 1
*f*(*X*)	0.9702	0.9939	1.0236	1.2328
R_BW_	1.4506	1.4875	1.7913	2.0828
CA_test_	0.97	0.965	0.935	0.905
